# Population Genomic Analysis of Two Endemic Schizothoracins Reveals Their Genetic Differences and Underlying Selection Associated with Altitude and Temperature

**DOI:** 10.3390/ani10030447

**Published:** 2020-03-07

**Authors:** Tianyan Yang, Wei Meng, Baocheng Guo

**Affiliations:** 1 College of Fishery, Zhejiang Ocean University, Zhoushan 316022, China; hellojelly1130@163.com; 2 Marine Fisheries Research Institute of Zhejiang, Key Laboratory of Sustainable Utilization of Technology Research for Fisheries Resources of Zhejiang Province, Scientific Observing and Experimental Station of Fishery Resources for Key Fishing Grounds, Ministry of Agriculture, Zhoushan 316021, China; 3 Key Laboratory of the Zoological Systematics and Evolution, Institute of Zoology, Chinese Academy of Sciences, Beijing 100101, China; 4 University of Chinese Academy of Sciences, Beijing 100049, China

**Keywords:** *Diptychus maculates*, *Gymnodiptychus dybowskii*, SLAF, genetic structures, adaptive evolution

## Abstract

**Simple Summary:**

*Diptychus maculates* and *Gymnodiptychus dybowskii* are two rare aboriginal fishes in the Xinjiang Uygur Autonomous Region. In recent years, due to overfishing and habitat fragmentation caused by construction of water conservancy and hydropower projects, the fishery resources have decreased sharply. Understanding the genetic background is of great significance for resource protection. In this study, we revealed the similar trends of population genetic diversities in these two species collected from the Tarim River and the Yili River. In addition, outlier SNPs associated with temperature and altitude were detected in both of them, indicating that Schizothoracinae fishes represented by *D. maculates* and *G. dybowskii* were still under the selection pressure of plateau environments.

**Abstract:**

Schizothoracins are a group of cyprinid fishes distributed throughout the Qinghai–Tibet Plateau, which can be classified in three grades: primitive, specialised and highly specialised according to adaptation ability to plateau environments. As the only specialised schizothoracins in Xinjiang, China, *Diptychus maculates* and *Gymnodiptychus dybowskii* are ideal materials for adaptive evolution research. Based on single-nucleotide polymorphism (SNP) loci detected by specific-locus amplified fragment (SLAF) technology, the genome-wide genetic diversities of these two species from nine sites in Xinjiang were evaluated. *D.maculates* in the Muzat River (BM) and *G. dybowskii* in the Kaidu River (LKG) presented the lowest genetic diversity levels, whereas *D. maculates* in the Kumarik River (BK) and *G.dybowskii* in the Kashi River (LK) were just the opposite. Cluster and principal component analysis demonstrated a distant genetic affinity between *D. maculates* in the Tashkurgan River (BT) and other populations. Outlier SNP loci were discovered both in *D. maculates* and *G. dybowskii*. The coalescent Bayenv and latent factor mixed model (LFMM) methods showed that a total of thirteen and eighteen SNPs in *D. maculates* were associated with altitude and temperature gradient, respectively. No intersection was revealed in *G. dybowskii.* The results indicated that *D. maculates* was subject to much greater divergent selection pressure. A strong signal of isolation-by-distance (IBD) was detected across *D. maculates* (Mantel test, rs = 0.65; *p* = 0.05), indicating an evident geographical isolation in the Tarim River. Isolation-by-environment (IBE) analysis implied that temperature and altitude selections were more intensive in *D. maculates*, with greater environmental variation resulting in weak gene flow.

## 1. Introduction

Ecological adaptation is the process in which organisms transform their shape, structure, physiological and biochemical characteristics based on changes in ecological environment factors. Ecological adaptation is accompanied by natural selection; it results in the survival of individuals with higher fitness. Environmental adaptation and incompatibility selection is a pair of contradictions manifested during speciation and evolution [1]. Native organisms have been well adapted to local climate and environment during thousands of years of evolution. Populations are locally adapted when they feature the highest relative fitness at their home sites and lower fitness in other parts of the range [2]. Studies on local adaptation provide important insights concerning the power of natural selection over gene flow and other evolutionary forces [3].

Xinjiang has complicated and unique geographical environments and various climate types. “Three mountains (Kunlun, Tianshan and Altai mountains) sandwiched between two basins (Tarim and Junggar Basins)” is the typical physiognomy conformation of Xinjiang, whereas hilly and basin areas are its two most common terrains [4]. Himalaya mountains, as a natural barrier, block the warm and wet airflow from the Indian Ocean, causing the cold, arid climate of the Qinghai Tibet Plateau (QTP), which also affects Northwest China. As China’s largest inland basin on the northern edge of QTP, the continental dry climate of Tarim Basin is a result of this phenomenon. Yili River Valley is located in the westerlies of high latitude. The warm and wet airflow from the distant Atlantic absorbs water vapours from several lakes in the Mediterranean; when it moves to Xinjiang and reaches the Yili Valley, the air climbs up and eventually condenses into raindrops, forming the warm and wet climate of Yili River Basin. The Tianshan Mountains can prevent air from transgressing and becomes the climate dividing line: northern Xinjiang belongs to the middle temperate zone, whereas southern Xinjiang belongs to the warm temperate zone [5,6]. All of these factors lead to the diversified water temperatures of various river systems in Xinjiang.

Schizothoracins are a group of cyprinid fish distributed throughout the QTP. Cao et al. divided Schizothoracinae into three evolutionary grades, namely, primitive, specialised and highly specialised grade, according to differences in habitat elevation, type of scale, pharyngeal teeth and barbells [7]. The changes in morphological and anatomical features result from adaption to a plateau environment during evolution. The scale coverage and number of pharyngeal teeth and barbels show a gradual decreasing trend with increasing altitude, that is, from the primitive to the highly specialised grade [7,8,9,10]. *Diptychus maculates* and *Gymnodiptychus dybowskii* are the only specialised schizothoracins in Xinjiang, China. Their distribution areas include Tianshan Mountains, Kunlun Mountains, Pamir Plateau, Tarim Basin and Yili Valley, leading to a changeable altitude [11]. Therefore, they are appropriate subjects for studying the adaptive evolution of Schizothoracine fishes [12,13]. The body of *G. dybowskii* is almost naked, with shoulder, anal and lateral scales only [11]. The situation of *D. maculates* is relatively complicated, with morphological variations existing in different geographical locations. In the Yili River Basin, the body scales of *D. maculates* are completely covered. However, in the Tarim River Basin, *D. maculates* exhibit partly squamate body scales, excluding those in Tashikuergan River, in which the upper and lower lateral scales have completely disappeared [11,14].

Does the environmental heterogeneity cause the population differentiation of these two species? In previous works, we have conducted molecular phylogeography studies of four fish species in the Tarim and Yili rivers and found a specific genetic diversity pattern among fishes [15,16,17]. Extensive genetic variations exist in *Schizothorax biddulphi* and *D. maculates* in the Tarim River, whereas the differentiations in *S. pseudaksaiensis*, *D. maculates* and *G. dybowskii* in the Yili River were relatively small. This phenomenon possibly resulted from the distance between the Tarim River and Qinghai–Tibet Plateau, which largely affected the uplift of the plateau and other geological movements. Genetic information based on a single or a few genes is limited, and only a small part of the evolutionary history or genetic structure can be revealed. With the implementation of the “Human Genome Project,” the development of next-generation sequencing technology enabled the development of molecular markers on a large scale. A series of cost-effective technologies represented by “reduced-representation sequencing” emerged. Specific locus amplified fragment (SLAF) is a set of “reduced-representation sequencing” technologies characterised by genotyping accuracy and marker efficiency [18]. Through one-time sequencing, SLAF technology obtains tens of thousands of molecular markers that evenly cover the entire genome. It can develop massive simple sequence repeat (SSR) and single-nucleotide polymorphism (SNP) markers at a lower cost, overcoming the one-sidedness of existing molecular markers [19]. Using the genome-wide SNPs obtained from the SLAF-seq, we first described the pattern of genetic variation distribution by investigating population structure and genetic diversity among *D. maculates* and *G. dybowskii*. Then, we tested whether population genetic differentiation within each species was associated with temperature and altitude gradient through detection of associated SNPs and isolation-by-environment (IBE).

## 2. Materials and Methods

### 2.1. Fish Materials

**Figure 1:**
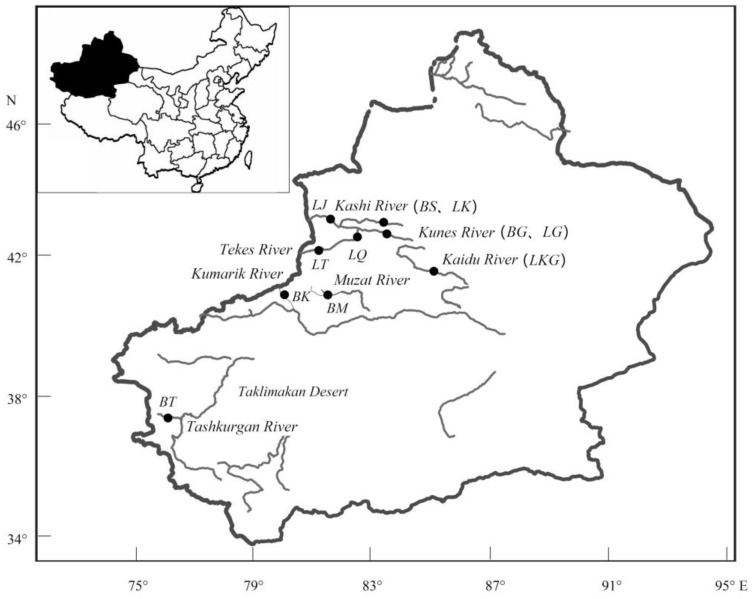
Sampling map of two Scizothoracins corresponding to Table 1.

According to the existing references [11,20,21,22] combined with the fishery resources investigations, we tried to collect samples in the distribution areas of Xinjiang as much as possible. A total of 124 specimens, including 57 samples of *D. maculates* from five locations and 67 samples of *G. dybowskii* from six locations, were collected in 2016 (Table 1, Figure 1). These sampling locations were basically covering the representative distribution areas of two species. Experimental procedures were approved by the Ethics Committee for Animal Experimentation of Zhejiang Ocean University (ECAE201816). The annual average environmental temperatures were obtained from Xinjiang fishery environment monitoring center and Fishery Resources and Environmental Science Experimental Station of Northwest China, Ministry of Agriculture. The altitudes were measured using a portable GPS device (GPSmap 631sc, Garmin, Taiwan).

### 2.2. DNA Extraction, SLAF Library Construction and High-Throughput Sequencing

Genomic DNA was extracted from muscle tissue using the standard phenol-chloroform method [23]. DNA quality was first assessed using 1% agarose gel and was further quantified using a NanoDrop^®^ ND-1000 spectrophotometer (Thermo Scientific, Waltham, MA, USA). SLAF sequence strategy with specific modifications was utilised in library construction. Briefly, the reference sequence of *Cyprinus carpio* (GenBank accession number: GCA_000951615.2) [24] was used to conduct the pre-experiment on silico simulation of the number of markers generated by various endonuclease combinations. The SLAF library was constructed based on the SLAF pilot experiment in accordance with the predesigned scheme, and eventually two endonuclease combinations of HaeIII and Hpy166II (New England Biolabs, Ipswich, MA, USA) were applied to genomic DNA digestion in the fish populations. Zhang et al. described the details of the SLAF sequence strategy [25]. The sequencing data were generated using Illumina HiSeq2500 platform. Raw data had been submitted to the National Center for Biotechnology Information (NCBI) Sequence Read Archive with the Bioproject number PRJNA599030.

### 2.3. SLAF Markers and SNP Calling

SLAF markers were identified and genotyped according to procedures described by Sun et al. and Zhang et al. [18,25]. All pair-end reads were clustered based on sequence similarity, and sequences with the identity over 95% were grouped to one SLAF locus. SLAF tags with sequence differences between samples can be defined as polymorphic SLAF tags, which allow for the development of specific molecular markers. The highest depth sequence in each SLAF tag was used as the reference sequence, and Burrows-Wheeler Aligner (BWA 0.7.17) software [26] was employed to align the sequencing reads to the reference genome under the default settings. SNPs were developed with the Genome Analysis Toolkit (GATK) [27] and SAMtools [28] methods. SNP filtering parameters were set as follows: (i) the minimum read depth was no less than 10; (ii) the average base quality was greater than 30. The intersection obtained using the two methods were the final SNP datasets (2,386,271). In order to establish an informative SNP panel, the high-quality SNPs were subjected to a filtering step with integrity of each SNP more than 0.6 and minor allele frequency (MAF) more than 0.05 as thresholds. As a result, a total of 124,441 SNPs were retained and used for further genetic analysis.

### 2.4. Data Processing

The populations program in Stacks 2.4 software pipeline was used to analyse the observed heterozygosity (*Ho*), expected heterozygosity (*He*), nucleotide diversity (*Pi*) and pairwise *Fst* [29]. SNP data were transformed to Plink format using Vcftools 0.1.14 [30] software and were exported to Plink 1.9 [31] software to conduct principal component analysis (PCA), whereas R 3.6.2 software was used for mapping. PGDSpider 2.1 [32] was employed to transform the SNP data to MEGA format; then, the neighbor-joining phylogenetic tree with bootstrapping method (1000 replicates) was constructed using MEGA 4.0 [33].

All SNPs were exported in GenePop format to BAYESCAN 2.1 to estimate the posterior probability that a given locus was affected by directional (positive alpha coefficient) or balancing selection/purifying selection (negative alpha coefficient) [34]. We applied a Bayesian approach as implemented in Bayenv 2.0 to remove outliers, so as to test for association between genetic differentiation and environmental parameters related to altitude and temperature gradient [35]. We used the Bayesian Markov chain Monte Carlo (MCMC) method and did the analyses on 20 pilot runs of 5000 iterations, followed by 50,000 iterations with a burn-in length of 50,000 iterations. Furthermore, in order to reduce the possible rate of false-positive, we validated the Bayenv results using a Latent Factor Mixed Model (LFMM 2.0) implemented in the R programing environment [36]. For LFMM analysis, the MCMC algorithm was used for environmental variables, using 1000 sweeps for burn-in and 10, 000 additional sweeps to calculate the |z|-scores for all SNP loci. The significance was tested using standard Gaussian distribution and Bonferroni correction for multiple testing. The cutoff |z|-score > 5 (*p* < 10^−7^) corresponded to a standard Bonferroni correction for a normal value type I error *α* < 0.01 and number of loci *L* = 10^5^.

The isolation-by-distance (IBD) test, which was first proposed by Wright in 1943, is the most common method for spatial genetic structure assessment [37]. It is defined as a decrease in genetic similarity among populations with increasing geographic distance. To test for IBD, we compared linearised *F_ST_* values [*F_ST_*/ (1 − *F_ST_*)] with log-transformed geographic distance using the Mantel test in the R package ‘ADE4’ [38]. For each of the Mantel test hypothesis combinations, we calculated the statistical significance through corrected permutation tests (*N* = 1000). We further tested for isolation-by-environment (IBE) by comparing pairwise genetic distance matrices with pairwise environmental variables (altitude and average annual temperature), with Mantel tests using the package ‘ADE4’ in the R environment [39]. To rule out potential false-positive correlations stemming from IBD effects, we conducted IBE tests by holding geographic distances constant with partial Mantle tests using the R package ‘VEGAN’ [40].

## 3. Results

A total of 217.54 M reads were obtained with the average Q30 89.98% and the average GC content 39.24%. A total of 836,213 SLAF-tags (average sequencing depth of 14.02×) were developed, in which 377,659 were polymorphism SLAF-tags. The range of SNPs numbers in every location were 751,073–724,167 (BM), 639,788–756,447 (BS), 722,348–804,217 (BK), 644,272–766,526 (BG), 635,865–682,990 (BT), 613,332–613,332 (LKG), 63,378–920,365 (LT), 812,056–933,336 (LQ), 761,654–937,587 (LK), 64,928–895,787 (LG) and 731,050–832,390 (LJ), respectively. Based on SNP loci, the genetic diversity level of two species at the whole genome level was evaluated and calculated (Table 2). The observed heterozygosity (*Ho*) of *D. maculates* and *G. dybowskii* ranged from 0.1549 to 0.2948 and from 0.1323 to 0.2805, respectively. The expected heterozygosity (*He*) ranged from 0.0961 to 0.2146 in the former and from 0.1071 to 0.2131 in the latter. Nucleotide diversity (*Pi*) is commonly used to measure the degree of polymorphism within a population. In this study, the maximum *Pi* was 0.2313 (BK), whereas the minimum value of 0.1282 (BM) was observed among *D. maculates* populations. In *G. dybowskii*, the genetic parameter ranged from 0.1146 to 0.2327. Inbreeding coefficient (*Fis*) of *D. maculates* varied from −0.0401 to −0.1257 and −0.0328 to −0.0961 in *G. dybowskii.* In general, the genetic diversities of two species showed that BM and LKG exhibited the lowest levels, whereas BK and LK showed the highest.

**Figure 2:**
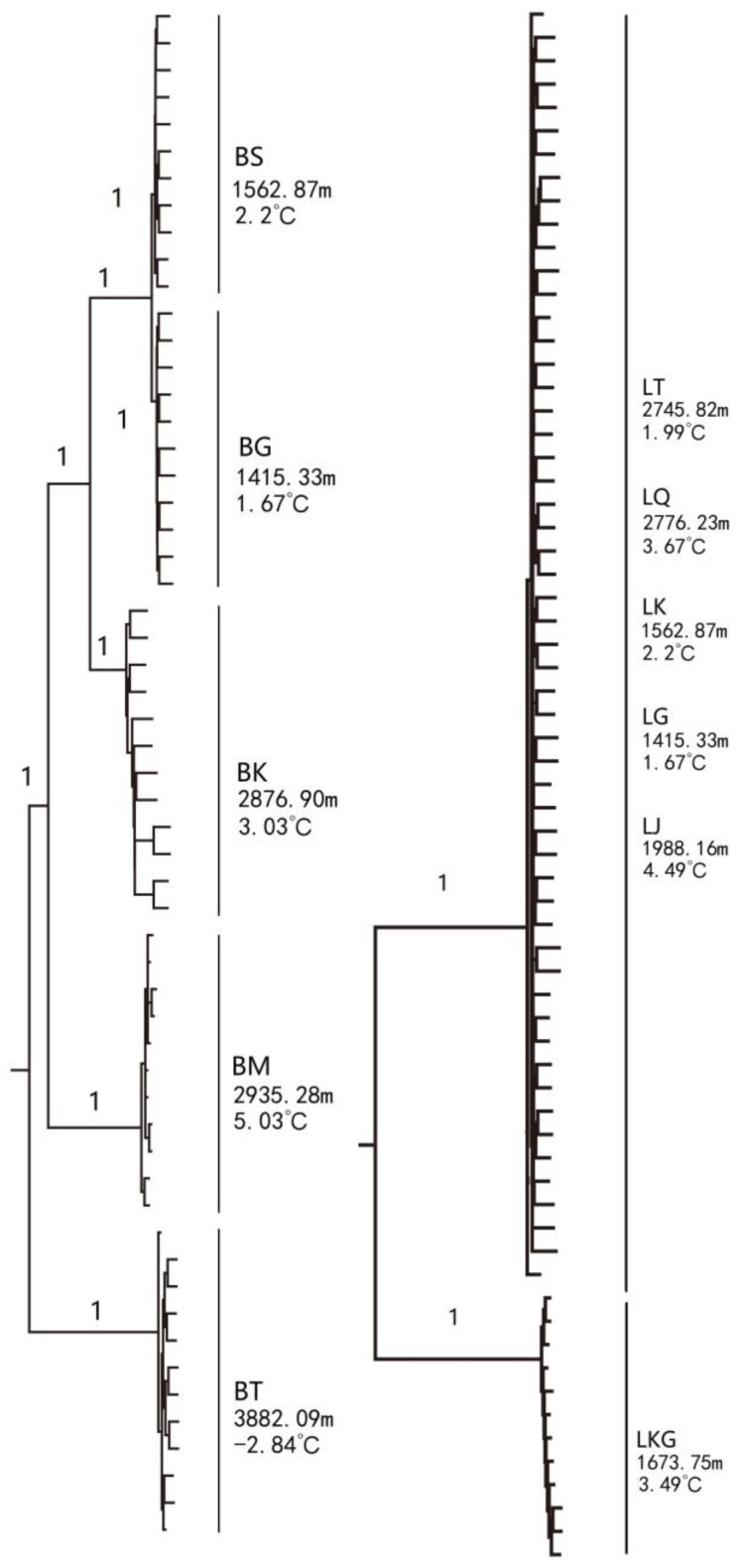
Phylogenetic trees of *D. maculates* (**left**) and *G. dybowskii* (**right**) constructed using the neighbor-joining (NJ) method (left—*D. maculates*; right—*G. dybowskii*). The node value means bootstrap support frequency.

The phylogenetic tree of *G. dybowskii* presented two major clades. Specimens of LKG were clustered under one clade, and the rest were aggregated to another with a high confidence value (99%) (Figure 2). The relationship among five *D. maculates* populations was relatively complicated. The dendrogram showed that except for BS and BG clustering together, all other populations (BT, BK and BM) formed distinct branches (Figure 2).

**Figure 3:**
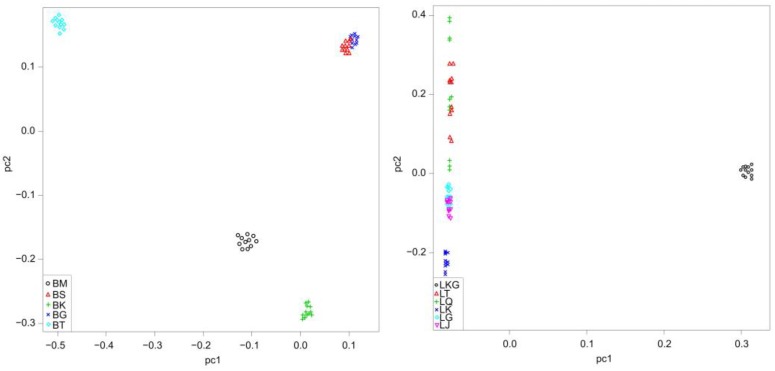
Principal component analysis (PCA) of *D. maculates* (**left**) and *G. dybowskii* (**right**) based on genome SNP data. The vertical line represents false discovery threshold of 0.01.

A PCA plot that was obtained based on genome-wide SNPs, revealed a geographically ordered population structure (Figure 3). The percentage of variance explained by the first principal component (PC 1) was 27.20% in *D. maculates* and 56.76% in *G. dybowskii*, while the second principal component (PC 2) explained 20.56% in *D. maculates* and 2.51% in *G. dybowskii* of the total variance, respectively. LKG, which was located in the positive abscissa axis, could be distinguished from other *G. dybowskii* populations using linear arrangement on the first principal component. Considering the second principal component of *D. maculates*, BT, BG and BS were in the forward direction of the second axis, whereas BM and BK were in the opposite direction. BG and BS clustered together, indicating their relatively close genetic relationships.

**Figure 4:**
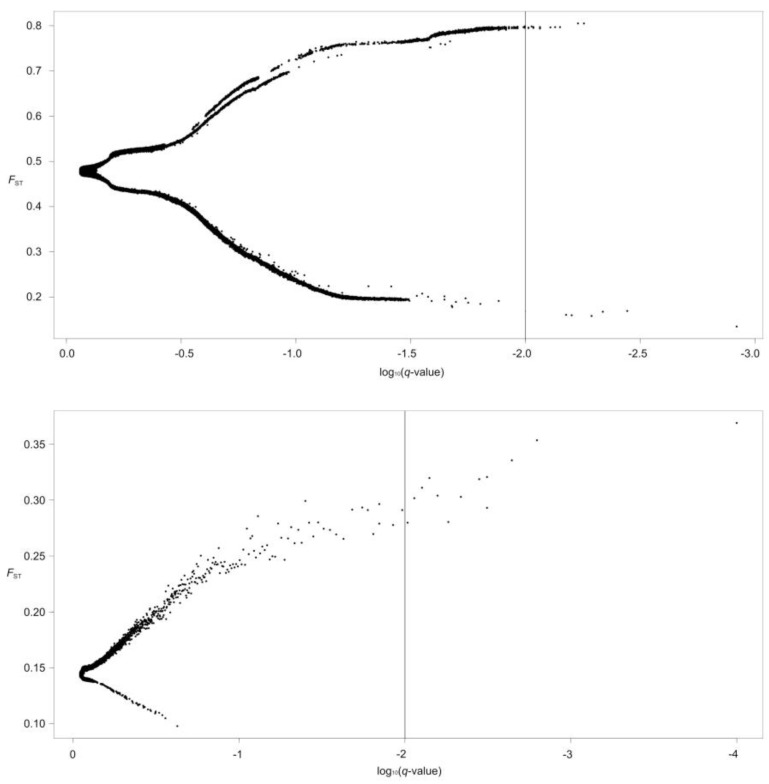
Global outlier detection based on 226,294 (*D. maculates,* **above**) and 271,307 (*G. dybowskii,* **below**) SNPs.

Global outlier detection based on 226,294 SNPs (*D. maculates*) and 271,307 SNPs (*G. dybowskii*) was performed using BayeScan (Figure 4). The average alpha value in *G. dybowskii* ranged from 2.0727 to 2.8024 with the average value 2.3687, suggesting that *G. dybowskii* may have experienced diversifying selection. In *D. maculates,* there were six negative alpha values, ranging from −2.0643 to −1.7605, with an average of −1.8579; and fourteen positive alpha values, ranging from 1.6680 to 1.7488, with an average of 1.6897. The result showed that both diversification selection and purification selection existed in *D. maculates*. Twenty outlier SNP loci were detected in *D. maculates* and thirteen outlier SNP loci were detected in *G. dybowskii*. Based on the criterion of a log10 Bayes factor greater than 0.5, 14,041 SNPs in *D. maculates* and 10 SNPs in *G. dybowskii* associated to altitude adaptation were found using the Bayenv method, respectively. In terms of temperature factor, 14,357 SNPs were revealed in *D. maculates* against 12 SNPs in *G. dybowskii.* Overall, 13,746 SNPs of *D. maculates* were related to temperature and altitude factors, whereas 2 SNPs in *G. dybowskii* were related to both factors. LFMM is an effective algorithm for estimating confounding factors and for testing gene-environment association (GEA), and decreased the number of false-positive associations in genome scans [41]. For this analysis, five major genetic clusters (*K* = 5) were chosen in *D. maculates* and two (*K* = 2) were applied in *G. dybowskii* based on the population structure analysis. A total of 1684 SNPs (1569 associated to temperature and 115 associated to altitude) were tested in *D. maculates* compared with only 22 SNPs (4 associated to temperature and 18 associated to altitude) in *G. dybowskii*. In general, thirteen shared SNPs between Bayenv and LFMM were associated with altitude gradient and eighteen loci were related to temperature gradient in *D. maculates*, separately. However, no intersection was detected in *G. dybowskii*.

Mantel tests present a straightforward method to examine both IBD and IBE of different SNPs using the R package (Table 3). The significant signal of IBD was detected across *D. maculates* (Mantel test, r_s_ = 0.65, *p* = 0.05) populations using Mantel correlations. The results demonstrated a positive correlation between *Fst* and geographic distance among *D. maculates* populations. IBE presented a correlation between genetic divergence and environmental dissimilarity. The pairwise *Fst* (Table 4) genetic and environmental distances were only significantly positive in *D. maculates* according to the Mantel test. There was significant population differentiation among *G. dybowskii* other than LKG (Table 5). The results of a partial Mantel test also suggested that correlation of genetic differentiation with altitude (r_s_ = 0.06) and temperature (r_s_ = 0.74) in *D. maculates* was higher than that in *G. dybowskii* (altitude r_s_ = −0.35, temperature r_s_ = 0.18).

## 4. Discussion

As an important part of biodiversity, genetic diversity is the basis of species diversity and ecosystem diversity. It is the product of long-term evolution and is the premise of survival adaptation [42,43]. Mitochondrial DNA (mtDNA), an effective molecular marker, has been widely applied for genetic diversity and phylogeographical pattern assessment within many vertebrate taxa [44]. BK population featured the highest genetic diversity in *D. maculates*. The results on mtDNA (*Cyt b*, *COI* and *D-loop*) revealed that the BK population included two branches: one was close to the Yili River, and the other branch was close to the Tarim River populations [16,45]. By contrast, only one clade was discovered in a dendrogram based on SLAF-seq. The incongruence might be caused by incomplete lineage sorting (ILS) during the phylogenetic history of *D. maculates.* However, the BK population were located at an intermediate position between the Yili River and the Tarim River in PC1 of a PCA plot, which presented consistency with mitochondrial results. Genetic recombination is one of the most important processes for biodiversity and can produce variation by inducing genetic diversity [46]. It was speculated that possible involvement of secondary contact occurred within the BK population, which reestablished gene flow between formerly diverged subpopulations and induced genetic introgression and recombination [47]. Thus, we proposed the hypothesis that genetic introgression or recombination of differentiated populations increased diversity of the BK population. The LKG population featured the lowest genetic diversity and showed large divergence from other *G. dybowskii* populations. Biogeographical evidence indicated that speciation of *G. dybowski* in Xinjiang occurred in the Tianshan Mountains. During the Quaternary interglacial period, it spread to the Kaidu River from the upper tributaries of the Yili River system in today’s Xinyuan, Gongliu and Hejing [48]. As the stenochoric species besides *Aspiorhynchus laticeps* in the Tarim River system, *G. dybowskii* only distributes in middle and upper reaches of the Kaidu River, a tributary of the Tarim River system, and has less communication with other populations [11,20]. Genetic drift denotes the change in frequency of an existing gene variant in a population due to random sampling of organisms [49]. The effect of genetic drift becomes stronger in a smaller population. It was speculated that the LKG population originated from a small group and experienced the “Founder Effect”, which was a special case of genetic drift and meant that when a population was established and developed by only a few individuals, the genetic information carried by these individuals cannot fully reflect the genetic information of the original population, resulting in low genetic diversity of the new population [50]. However, the *G. dybowskii* species were widely distributed in the Yili River system, with each population being relatively independent, whereas certain genetic recombination among the Yili River populations might result in a high genetic diversity.

The emergence of specialised Schizothoracinae represents a specific historical stage in the uplift process of the Qinghai–Tibet Plateau. The different habitat altitudes reflect the differences in the environmental conditions that fishes adapt to. The habitat elevation of specialised Schizothoracinae was lower compared with the highly specialised Schizothoracinae but higher than the primitive Schizothoracinae. Only primitive and specialised Schizothoracinae fish are evident in Xinjiang. This finding might be related to the notable distance of Xinjiang from the Qinghai–Tibet Plateau, which was minimally affected by the uplift. The mountains in Xinjiang showed no uplift to a higher altitude in a wide range. Cluster analysis of *D. maculates* located the BT population at the base of the phylogenetic tree. Principal analysis also displayed the distant plot of BT from the other groups. This finding is possibly attributed to the location of the BT population, which was solely found at the south corner of the Tarim Basin with a high altitude. Evolutionary adaptation may occur when wildlife populations migrate to new environments for multiple generations [51]. The fishes of Tashikurgan firstly adapted to Pamir plateau environment and formed an independent population through evolutionary selection. *D. maculates* in Tashikuergan notably lack lateral scales, suggesting that the population is under strong plateau environment selection pressure. A low number of scales may be associated with low oxygen content in the plateau environment. The bare skin was conjectured to contribute to respiration, similar to loach, which is a hypoxia-tolerant fish with missing scales. The BM and BK population also formed their relatively independent clades, which reflected the geographical isolation of the Tarim River. However, the plots of the BM and BK population were close, possibly because the two populations were located at the north margin of the Tarim Basin. The two populations of the Yili River (BG and BS) gathered to form one clade, which indicated a higher gene exchange in the Yili River than in the Tarim River. The close relationship between the populations in principal analysis also proved this point, consistent with previous study results of *S. biddulphi* and *S. pseudaksaiensis*. The phenomenon can be explained by the following reasons. The geographic distances of the Yili River tributaries were close and concentrated, the Tarim River system populations lie distantly from each other and the stream was often interrupted by desert barriers.

The phylogenetic tree of *G. dybowskii* formed two branches, with the LKG population differing from other populations in the Yili River. In principal analysis, the Yili River group overlapped on the left side, whereas the LKG population was located on the right side, which also confirmed that LKG was divergent from other populations. The Kaidu River belonging to the Tarim River system is separated from Yili River by the Tianshan Mountains. The two populations could not link with each other or lacked genetic communication. The phylogenetic analysis revealed that the gene exchange in the Yili River was highly similar to that of *D. maculates*. The principal analysis further showed that LT and LQ and LG and LJ crisscrossed each other. On the geographical distribution map, these pairs were located in the same tributary separately. LT and LQ were located on the south bank of the Yili River and LG and LJ were located midstream of the Yili River, whereas LK was located on the north riverbank alone.

Natural selection causes allele frequency changes in a large population, leading to genetic evolution over evolutionary time scales. Natural selection is a critical driving force of biological evolution. Individuals with different phenotypes and genotypes exhibit different adaptabilities to specific natural environments in a population [52]. Altitude adaptation refers to the adaptation acquired by natural selection of animals living on the plateau for thousands of years and features a genetic basis [53,54]. Schizothoracinae is a representative species that adapts to the environment around the Qinghai–Tibet Plateau [7]. Investigating the effect of natural selection on Schizothoracinae fishes is important to study plateau adaptation and evolution. BayeScan aims to identify candidate loci under natural selection from genetic data, using differences in allele frequencies between populations [55]. In the results, outlier SNP loci were discovered in both *D. maculates* and *G. dybowskii*, indicating that Schizothoracinae is still under the selection pressure of plateau environments. However, the outlier SNP loci accounted for a minimal proportion of the total SNP loci, possibly because the Qinghai–Tibet Plateau has undergone a rapid uplift period and is in a relatively stable stage nowadays.

Background genetic variation along environmental gradients has been documented among and within many species; Bayenv is a relatively new method developed for detecting patterns of polymorphisms associated to environmental gradients [56]. As Schizothoracinae evolved from a group of fishes adapting to a plateau environment, its genetic variation likely exhibited a certain connection with altitude. Schizothoracinae include cold-water fish, and the temperature will also change as elevation increases. Therefore, temperature might be another environmental factor associated to genetic background. In this study, numerous SNP loci associated to altitude and temperature were detected in *D. maculates*, whereas fewer loci were found in *G. dybowskii* using two methods (Bayenv and LFMM). This indicates that *D. maculates* was under greater divergent selection pressure. During the sampling process, we observed that the altitude of *D. maculates* was higher than *G. dybowskii*. *D. maculates* can better adapt to higher altitude than *G. dybowskii*, which can be explained by its more relevant SNPs.

Qualitative and statistical analyses of isolation-by-distance or environment can reveal information about population genetic structure [57]. IBD and IBE analyses determine how strongly genetic differentiation between populations is driven by geographical distance and environmental differences. The primary use for (genetic) isolation by (geographic) distance is to assess whether more distant population pairs are more genetically different [57]. In this study, the IBD test of *D. maculates* yielded significant results, showing a notable geographical isolation in the Tarim River. The extent of local adaptation was determined through the balance between gene flow and selection [2]. Gene flow may follow patterns of IBE, whereby gene flow rates are high among similar environments [58]. The Mantel test showed that genetic isolation of *D. maculates* was significantly correlated with temperature, and the *P* value of altitude was small in *D. maculates.* Moreover, the spearman rank correlation coefficient (r_s_) value of *D. maculates* was also greater than that of *G. dybowskii*. Meanwhile, r_s_ values of altitude and temperature in *D. maculates* were also higher in partial Mantel statistics. The divergent selection of temperature and altitude was likely more intensive in *D. maculates*, with the greater environment variation resulting in its weak gene flow.

## 5. Conclusions

Extensive genetic variations existed in *D. maculates* and *G. dybowskii* of the Tarim River and YIli River, which might be caused by the special topography in the Tarim basin, complex water system distribution and the barrier effect of the Tianshan Mountains. Natural selection tests at the genome-wide level revealed obvious local adaptation to temperature and altitude. The results of this study would help to provide basic references for Schizothoracinae germplasm resource protection in Xinjiang.

## Figures and Tables

**Table 1:** Sampling information of two Scizothoracins.

Species	Population	River	River Basin	Sample Size	Altitude (m)	Temperature (°C)
*Diptychus maculates*	BM	Muzat River	Tarim River	11	2935.28	5.03
	BK	Kumarik River		12	2876.90	3.03
	BT	Tashkurgan River		12	3882.09	−2.84
	BG	Kunes River	Yili River	11	1415.33	1.67
	BS	Kashi River		11	1562.87	2.2
*Gymnodiptychus dybowskii*	LKG	Kaidu River	Tarim River	12	1673.75	3.49
	LQ	Qiapugihai Reservoir	Yili River	11	2776.23	3.67
	LK	Kashi River		11	1562.87	2.2
	LG	Kunes River		11	1415.33	1.67
	LJ	Yamadu		11	1988.16	4.49
	LT	Tekes River		11	2745.82	1.99

**Table 2:** Genetic diversity indices for two Scizothoracins calculated using genome single-nucleotide polymorphism (SNP).

Population	Obs Het	Exp Het	*Pi*	*Fis*
BM	0.1549	0.0961	0.1282	−0.0401
BS	0.2439	0.1750	0.1912	−0.1017
BK	0.2948	0.2146	0.2313	−0.1257
BG	0.2419	0.1735	0.1895	−0.1007
BT	0.1789	0.1191	0.1429	−0.0604
LKG	0.1323	0.1071	0.1146	−0.0328
LT	0.2687	0.2115	0.2311	−0.0742
LQ	0.2616	0.2097	0.2293	−0.0635
LK	0.2805	0.2131	0.2327	−0.0961
LG	0.2622	0.2069	0.2261	−0.0712
LJ	0.2554	0.2059	0.2251	−0.0584

**Table 3:** Isolation-by-distance (IBD) and isolation-by-environment (IBE) tests with SNP data for two Scizothoracins.

	IBD		IBE							
			Altitude				Temperature			
			Mantel Test		Partial Mantel Test		Mantel Test		Partial Mantel Test	
	r_s_	*p*	r_s_	*p*	r_s_	*p*	r_s_	*p*	r_s_	*p*
*D. maculates*	0.65	0.05	0.42	0.09	0.06	0.47	0.92	0.01	0.74	0.17
*G. dybowskii*	0.50	0.10	−0.12	0.75	−0.35	0.92	−0.18	0.77	0.18	0.25

r_s_ = Spearman rank correlation coefficient, *p* refers to empirical significance level from 1000 permutations.

**Table 4:** The pairwise *Fst* values of different populations in *D. maculates*.

	BM	BS	BK	BG	BT
BM		0.28391	0.21963	0.28838	0.44715
BS	0.28381		0.17747	0.05600	0.32412
BK	0.21963	0.17737		0.18040	0.27498
BG	0.28827	0.05599	0.18029		0.32785
BT	0.44715	0.32404	0.27498	0.32776	

The numbers below the diagonal mean average *Fst* values exclude outlier SNPs and the numbers above the diagonal mean average *Fst* values include outlier SNPs. BM means Muzat River, BS means Kashi River, BK means Kumarik River, BG means Kunes River and BT means Tashkurgan River.

**Table 5:** The pairwise *Fst* values of different populations in *G. dybowskii*.

	LKG	LT	LQ	LK	LG	LJ
LKG		0.32159	0.32137	0.32192	0.32457	0.32348
LT	0.32158		0.04007	0.04102	0.04161	0.04117
LQ	0.32137	0.04006		0.04078	0.04166	0.04085
LK	0.32192	0.04101	0.04078		0.04136	0.04016
LG	0.32457	0.04160	0.04165	0.04135		0.04192
LJ	0.32347	0.04116	0.04085	0.04016	0.04192	

The numbers below the diagonal mean average *Fst* values exclude outlier SNPs and the numbers above the diagonal mean average *Fst* values include outlier SNPs. LKG means Kaidu River, LT means Tekes River, LQ means Qiapugihai Reservoir, LK means Kashi River, LG means Kunes River and LJ means Yamadu.
